# Editorial: Modulating lymphatic vascular growth in disease: current and potential pharmacological approaches for prevention and treatment, Volume II

**DOI:** 10.3389/fphar.2023.1224414

**Published:** 2023-07-04

**Authors:** Natalie L. Trevaskis, Ines Martinez-Corral, Melissa García-Caballero

**Affiliations:** ^1^ Department of Drug Delivery, Disposition and Dynamics, Monash Institute of Pharmaceutical Sciences, Monash University, Melbourne, VIC, Australia; ^2^ Laboratory of Development and Plasticity of the Neuroendocrine Brain, Lille Neuroscience & Cognition, Inserm UMR-S1172, University of Lille, Inserm, CHU Lille, Lille, France; ^3^ Department of Molecular Biology and Biochemistry, Faculty of Sciences, University of Málaga, Andalucía Tech, Málaga, Spain; ^4^ IBIMA (Biomedical Research Institute of Málaga)-Plataforma BIONAND, Málaga, Spain

**Keywords:** lymphatic vessels, lymphedema, cancer, treatment, models

The lymphatic system is crucial for the maintenance of the tissue fluid homeostasis, immune cell trafficking and the absorption of dietary lipids in the intestine (Alitalo, 2011; Oliver et al., 2020; Rockson, 2021). Therefore, failure of the lymphatic system leads to multiple diseases, including cancer, autoimmune diseases, cardiometabolic diseases, infection diseases, and more recently, neurodegenerative diseases (Oliver et al., 2020). Moreover, an impaired drainage capacity results in lymphedema, a chronic inflammatory disorder characterized by edema, chronic swelling, fat deposition and fibrotic tissue accumulation in the extremities (Rockson, 2021). Currently, no approved pharmacological treatment is available for this disorder, and interventions such as physiotherapy and compression garments, transiently reduce edema but are ineffective in preventing or reversing disease progression. Thus, there is not only an urgent need to further improve our understanding of the role of lymphatics in disease but to develop novel lymph-targeted therapies that improve the quality of life of the patients suffering from it. In this Research Topic, we present research articles about the lymphatic vasculature that provide novel insights into the impact of surgery and radiotherapy on lymphatic vasculature, the effect of doxycycline treatment in secondary lymphedema, a new cervical lymph method to assess lymphatic transport in rats; and a review article that highlights the hypoxia-regulated pathways in lymphedema.

In developed countries, secondary lymphedema occurs mostly as a consequence of cancer treatments that involve lymph node resection or radiotherapy (Rockson et al., 2019). Considering that no efficient therapy exists, new approaches and animal models are needed to better understand the cellular and molecular mechanisms underlying the pathophysiology of lymphedema. In the study of Buntinx et al. the authors describe a murine model that combines local irradiation of the murine hind limb together with lymph node resection in order to investigate their impact in lymphedema. The study highlights the importance of radiation prior to surgery to rapidly induce severe tissue remodelling that includes the different hallmarks of lymphedema including swelling (paw thickness), lymphatic vasculature remodelling, epidermal/dermal thickening and other histological features (i.e., organizational pattern of collagen fibers, the presence of non-structural matricellular proteins, *etc.*). Moreover, by using a computerized method of digital image quantification, Buntinx et al. established the spatial distribution of lymphatic expansion and matrix deposition in papillary and reticular dermis, describing for the first time, an excessive deposition of periostin and tenascin-C in an experimental model of lymphedema. This represents a novel mouse model to study the pathophysiology of lymphedema and test potential therapeutic targets.

In the context of radiation and surgical therapies for the treatment of cancer, Pillay et al. examine the molecular mechanisms underlying the damage on lymphatic vessels and lymphatic endothelial cells (LECs) that lead to the development of lymphedema upon these treatment modalities. To better decipher the impact of radiation on one of the main lymphatic signaling pathways, the VEGF-C/VEGF-D/VEGFR-3, they use different *in vitro* approaches to mimic the different cellular processes occurring during the lymphangiogenic process. They also use animal models of lymphatic injury. Their experiments show that radiotherapy selectively affects crucial LEC functions required for lymphangiogenesis such as proliferation and sprouting. This impairment is mainly mediated by the downregulation of VEGFR-3 after radiation exposition, and thus the lower response of LECs to the lymphangiogenic growth factors VEGF-C and VEGF-D. In conclusion, this study highlights the clinical limitations of using VEGF family lymphangiogenic growth factors for the treatment of lymphedema.

As mentioned above, there is no effective medical treatments for secondary lymphedema, and so far, only symptom-controlling physiotherapy exists (Rockson et al., 2019). Interestingly, data from a phase 1 clinical trial suggests that doxycycline is effective for the treatment of filariasis-induced lymphedema, however, no data about the effect of doxycycline for treating cancer-related lymphedema is available. In this Research Topic, Brown et al. use doxycycline in an off-label manner in patients who developed breast cancer-related secondary lymphedema between January 2021 and January 2022. In their work, patients with upper extremity lymphedema and a volume difference of >5% were orally treated with doxycycline (200 mg, administered once a day for 6 weeks), and although doxycycline had no significant effect on relative limb volume change or L-Dex scores, the authors report a significant improvement in patient-reported quality of life, due to improvements in the physical and psychological wellbeing subscales. While there are some limitations in this study, such as the small sample size, their results highly encourage the design of future trials, with larger sample size and more objective measures (i.e., ultrasound, trans-epidermal water loss, skin stiffness, skin water content, histology, *etc.*) to fully determine the role of doxycycline in cancer patients suffering from lymphedema.

Prior studies have shown that tumor-associated lymphangiogenesis is controlled by hypoxia, which triggers *Prox1* expression, the master regulator of the lymphatic vasculature, as well as the main lymphangiogenic growth factors, such as VEGF-C/VEGFR-3, and CXCL12/CXCR4 (Ji, 2014; Morfoisse et al., 2015). Moreover, a range of studies performed on preclinical models have revealed the presence of hypoxic areas with altered metabolite levels in lymphedematous tissue (Jiang et al., 2020), suggesting the important role of hypoxia in regulating lymphangiogenesis during lymphedema progression. This data is supported by clinical studies revealing the presence of inflammation in lymphedema tissues (Azhar et al.) and its crucial role for lymphedema progression (Azhar et al.). In this context, Jiang et al. review recent findings on the regulation of lymphedema by hypoxia and hypoxia-inducible factor (HIF)-mediated responses, and the impact of HIF isoforms (HIF-1α and HIF-2α) in lymphangiogenesis, inflammation, fatty expansion, and fibrotic tissue remodeling. Interestingly, it has been suggested that HIF isoforms possibly have opposing roles in lymphedema, with HIF-2α having beneficial effects. Increased HIF-1α expression with concomitant HIF-2α reduction in LECs may exacerbate lymphatic malfunctioning (Jiang et al.). On the other hand, both isoforms seem to have opposite roles in adipocytes, in certain situations such as obesity, where the accumulation of fat and metabolism is similar to that in lymphedema. Here, HIF 1α has a promoting effect and HIF-2α protects against metabolic maladaptation, insulin resistance and inflammation in diet induced obesity (Garcia-Martin et al., 2016). The authors conclude that, since tissue hypoxia and responses to hypoxic stress regulate immune function and fibrosis in several pathologies, a better understanding of the hypoxia-regulated molecular cascades will help us to gain insight into lymphedema pathogenesis and investigate novel therapeutic targets in this disease.

With no doubt, new approaches and methods for the study of the lymphatic vasculature and the lymph composition are needed. Hoang et al. present, for the first time, a novel method to collect efferent cervical lymph fluid from Male Sprague-Dawley Wistar rats through cannulation of the cervical lymph ducts to evaluate cervical lymph composition (i.e., lipids, proteins and immune cells). To do so, they cannulate the rats at the carotid artery with or without cannulation at the cervical lymph duct, and collect samples of blood, whole lymph and isolated lipoprotein fractions of lymph to determine lipid and protein composition, and to analyse by flow cytometry analysis the different cell populations present in the sample. Since lymphatic drainage pathways from the brain (and head) converge at the cervical lymphatics, collection of this lymph fluid allows the characterization of lymphatic transport of a range of metabo-lites and cells from the brain into lymph in physiological and pathological conditions. Additionally, one of the advantages of this technique compared to those described previously, is the collection of larger volumes of lymph, as well as the collection of efferent rather than afferent lymph, which might be interesting for the study of the different immune cell trafficking pathways, for instance. This new approach enables the quantitative analysis of changes in cervical lymph composition and transport in health and disease and could offer a broad range of applications in CNS research and the discovery of novel biomarkers and therapeutic targets.

In summary, this second edition of our article Research Topic, tackles once more the importance of finding new therapies for patients suffering from lymphedema. This covers not only from the clinical side with new clinical trials with possible pharmacological treatments, but also from the basic research side, where the development of new approaches and methods are essential to increase our knowledge on the mechanisms behind this pathology.
